# Correction: Unlocking the power of synergy: High-intensity functional training and early time-restricted eating for transformative changes in body composition and cardiometabolic health in inactive women with obesity

**DOI:** 10.1371/journal.pone.0305040

**Published:** 2024-05-31

**Authors:** Ranya Ameur, Rami Maaloul, Sémah Tagougui, Fadoua Neffati, Faten Hadj Kacem, Mohamed Fadhel Najjar, Achraf Ammar, Omar Hammouda

S1 Data File is omitted from the list of Supporting Information. It can be viewed below.

## Supporting information

S1 File Study data. This file includes supplementary data.
(PDF)

## References

[1] Ameur R, Maaloul R, Tagougui S, Neffati F, Hadj Kacem F, Najjar MF, et al. (2024) Unlocking the power of synergy: High-intensity functional training and early time-restricted eating for transformative changes in body composition and cardiometabolic health in inactive women with obesity. PLoS ONE 19(5): e0301369. 10.1371/journal.pone.0301369. 38691521 PMC11062533

