# Supplementary material for: Unlocking the power of synergy: High-intensity functional training and early time-restricted eating for transformative changes in body composition and cardiometabolic health in inactive women with obesity
Source: PLoS One. 2024 May 1;19(5):e0301369. doi: 10.1371/journal.pone.0301369 (PMC11062533; doi:10.1371/journal.pone.0301369)
Supplement: S1 Protocol — (DOCX) [file pone.0301369.s002.docx]

**Study Protocol**

**TITLE PAGE**

**Full Title** Unlocking the power of synergy: High-intensity functional training and early time-restricted eating for transformative changes in body composition and cardiometabolic health in inactive women with obesity

**Short Title/Acronym** TRE and HIFT

**Research Ethics Committee** C.P.P.SUD N ° 0200/2019

**Reference**

**Chief Investigator** Dr Omar Hammouda, Interdisciplinary Laboratory in Neurosciences, Physiology and Psychology: Physical Activity, Health and Learning (LINP2), UFR STAPS, UPL, Paris Nanterre, Nanterre, France.

**Mail** [Omar.hammouda@parisnanterre.fr](mailto:Omar.hammouda@parisnanterre.fr)

**INTRODUCTION**

**Background**

Obesity is an epidemic, recurrent and progressive pathological process with many causal factors (Bray et al.,2017). Obesity affects about 650 million people, or 13% of the world’s adult population (Haththotuwa et al.,2020). In this scenario, obesity represents a fundamental public health clinical problem, as it increases the risk of cardiovascular disease, insulin resistance, cancer, osteoarthritis (Haththotuwa et al.,2020) as well as complications of postural control and alterations in physical performance (Cahill et al.,2013; Gupta et al.,2017 ). For obese people, lifestyle changes such as changing eating behaviors and increasing physical activity are generally recommended and known to be major components of weight reduction programs (Bellicha et al.,2021). In recent years, time restricted eating (TRE) has emerged as an intermittent fasting diet and a potential alternative to continuous calorie restriction CCR (O'Connor et al., 2021), which has been shown to improve cardiometabolic health, insulin sensitivity, blood glucose levels, lipid profile, blood pressure (Rynders et al.,2019), as well as improved liver function, intestinal microbial diversity and circadian gene expression (Zeb et al.,2020), and decreases oxidative stress (Sutton et al.,2018). TRE becomes a popular strategy because it is easy to follow for a longer period of time and does not require people to limit their overall food intake or calculate total daily calorie intake, which can improve adherence (O'Connor et al.,2021).

Moreover, regular physical activity remains one of the most powerful treatments to combat obesity (Bellicha et al.,2021). The American College of Sports Medicine recommends at least 150 min/week of moderate intensity aerobic activity or 75 min/week of vigorous intensity to improve health, and 225 min/week or more is recommended for weight loss/maintenance (CMHA , 2013). However, compliance with these guidelines is low, and is a frequently cited obstacle (e.g., walking/running fast), which is perceived as repetitive and boring and takes time (ACSM, 2013; Ekkekakis et al.,2022). In order to reduce the time spent on exercise, high intensity interval training (HIIT) has recently gained popularity as a good exercise choice for obese people (Andreato et al.,2019). This type of exercise consists of short periods of high intensity activity followed by periods of rest or low intensity (Gillen et al., 2014; Andreato et al., 2019). Cycling, running and rowing are traditional exercise modalities that have adapted HIIT protocols, but for people who exercise for health and leisure, these traditional modalities seem boring and do not engage people because of the monotonous nature of the exercise associated with repetition (Sharp et al.,2022). An alternative to HIIT is high intensity functional training (HIFT), a relatively new training modality that includes a combination of aerobic exercise and resistance and consists of alternating short periods of intense exercise with periods of rest or active recovery of moderate intensity using movements multi-functional varied articular.

HIFT is generally performed in a circuit training format at high relative intensities, with the aim of simultaneously improving all physical attributes (Feito et al.,2018). In overweight/obese adults with type 2 diabetes mellitus, Nieuwoudt et al. (2017) showed significant improvement in beta cell function, while decreasing body fat and preserving lean mass after 6 weeks of HIFT. Similarly, Fealy et al.,2018) reported improvements in cardiometabolic risk factors with HIFT training. Research indicates that HIFT is superior to HIIT to improve health (Heinrich et al.,2014; Fisher et al., 2017). With respect to the combination of exercise and fasting, there are few studies that have investigated the combined effect of TRE and endurance/resistance training on body composition and performance parameters in healthy individuals (Moro et al.,2020; Tovar et al.,2021), but little is known about the metabolic benefits of this combined therapy in a sedentary overweight/obesity population.

**TRIAL OBJECTIVES**

**Primary Objective**

The primary objective of the study is to investigate the isolated and combined effects of 12 weeks of TRE and HIFT on weight and body composition in inactive obese women.

**Secondary Objectives**

The secondary objectives were to examine the effects of TRE and HIFT on changes in food intake, blood pressure, lipid profile, blood glucose, cortisol and inflammatory biomarkers, and to determine the feasibility of the combined approach (TRE + HIFT) by examining adherence to TRE and HIFT to inform any future randomized trials of the intervention.

**Primary Outcome**

Primary Outcome include the effect of the separate and combined effects of 12 weeks of early TRE (i.e., consuming all calorie intake between 8:00 a.m. and 4:00 p.m. daily) and HIFT (three exercise sessions per week), on body composition in women with obesity.

**Secondary Outcomes**

- Changes in blood pressure from baseline to 3 months
- Changes in lipid profile from baseline to 3 months
- Changes in glucose profile from baseline to 3 months
- Changes in calorie intake from baseline to 3 months
- Adherence to TRE and HIFT

**METHODOLOGY**

**Inclusion Criteria**

- Females aged 18–45 years old
- BMI higher than 30 kg/m^2^
- Waist circumferences higher than 80 cm
- Able to engage in physical activity
- Had not followed a structured training program in the previous 6 months
- Had maintained a constant weight for three months prior to the study

**Exclusion Criteria**

- Having a diagnosis of diabetes
- Having cardiovascular disease
- Smokers
- Using regular medications
- Pregnancy or breastfeeding
- Has lost more than 5% of body weight in the last 6 months
- Currently using TRE or another fasting approach to weight loss.
- Habitual eating window <12 hours/day

**STUDY DESIGN**

**Participant recruitment**

Participants will be recruited recruited using social media advertisements, leaflets and posters in community venues, and word of mouth.

Prospective participants will contact the study team by telephone or e-mail. They will be screened and if eligible, will receive an appointment for the baseline visit and participant information sheet will be sent by post or e-mail.

**Baseline Visit**

At the baseline visit, after completing the consent procedures, eligible participants will complete study questionnaires, and measurements of weight, blood pressure and fasting blood sample collection. Participants will than receive an explanation of the TRE and HIFT interventions and of the follow-up procedures.

**Randomization**

Participants will be randomized in a 1:1:1 manner to TRE, HIFT and TRE-HIFT groups using a web-based system for random number generation by a person independent of the research group. Participants in the combined group (TRE-HIFT) followed the same exercise protocol as per the HIFT group and the same TRE protocol as the TRE group.

**TRE intervention**

Participants will be asked to consume all their food during an 8-hour period (from 8:00 am to 4:00 pm) each day for the next three months. Over the remaining 16 hours each day, participants can drink, but only non-calorie drinks (e.g. water or coffee/tea with no milk or sugar).

**HIFT intervention**

HIFT will be performed 3 days per week in the evening at the fasting window (i.e. 5:00 p.m.). All HIFT sessions include 5 minutes of dynamic warm-up and 5 minutes of cool-down, in addition to 45-55 minutes of HIFT. Each HIFT session corresponds to 8 series of 8 functional exercises (aerobic and resistance) based on Tabata training. All exercises will be performed with body weight or with free weights (e.g. dumbbells, kettlebell, weight plate) using a self-selected pace and weight/resistance. Participants should perform as many repetitions as possible of a given exercise for 20 or 30 seconds, followed by a 10-second rest. There were 2 minutes of rest between each exercise.

**Measures**

***Baseline:***

- Demographic information collected via routine clinic questionnaires
- Weight, BMI
- Blood pressure
- Blood lipid profile and glucose level
- Physical activity levels (IPAQ)

***Final follow-up at 12 weeks:***

- Weight
- Blood pressure
- Blood Lipid profile and glucose level
- IPAQ
- TRE adherence

**Sample Collection, Labelling and Logging**

Blood samples analysis will be conducted by the Doctors laboratory at the University Hospital of Monastir (5000 Monastir, Tunisia).

Samples would be collected by trained phlebotomists in accordance with the Doctors Laboratory instructions, and labelled with participant ID number, time-point/session number, and date.

All samples taken will be logged.

**ETHICS**

The study was carried out in accordance with the Helsinki Declaration and was approved by the “South Human Protection Ethics Committee” (C.P.P.SUD), Sfax, Tunisia: protocol reference C.P.P.SUD N ° 0200/2019, prior to the beginning of the assessments.

**REFERENCES**

1. Haththotuwa RN, Wijeyaratne CN, Senarath U. Worldwide epidemic of obesity. Obesity and obstetrics: Elsevier; 2020. p. 3-8.

2. Bellicha A, van Baak MA, Battista F, Beaulieu K, Blundell JE, Busetto L, et al. Effect of exercise training on weight loss, body composition changes, and weight maintenance in adults with overweight or obesity: An overview of 12 systematic reviews and 149 studies. Obes Rev. 2021;22 Suppl 4(Suppl 4):e13256. <http://dx.doi.org/10.1111/obr.13256>. PMID: 33955140.

3. O'Connor SG, Boyd P, Bailey CP, Shams-White MM, Agurs-Collins T, Hall K, et al. Perspective: Time-Restricted Eating Compared with Caloric Restriction: Potential Facilitators and Barriers of Long-Term Weight Loss Maintenance. Adv Nutr. 2021;12(2):325-33. <http://dx.doi.org/10.1093/advances/nmaa168>. PMID: 33463673.

4. Rynders CA, Thomas EA, Zaman A, Pan Z, Catenacci VA, Melanson EL. Effectiveness of intermittent fasting and time-restricted feeding compared to continuous energy restriction for weight loss. Nutrients. 2019;11(10):2442. <http://dx.doi.org/10.3390/nu11102442>. PMID: 31614992.

5. Kirkham AA, Parr EB, Kleckner AS. Cardiometabolic health impacts of time-restricted eating: implications for type 2 diabetes, cancer and cardiovascular diseases. Curr Opin Clin Nutr Metab Care. 2022;25(6):378-87. <http://dx.doi.org/10.1097/MCO.0000000000000867>. PMID: 36017558.

6. Rothschild J, Hoddy KK, Jambazian P, Varady KA. Time-restricted feeding and risk of metabolic disease: a review of human and animal studies. Nutrition reviews. 2014;72(5):308-18. <http://dx.doi.org/10.1111/nure.12104>. PMID: 24739093.

7. Chow LS, Manoogian ENC, Alvear A, Fleischer JG, Thor H, Dietsche K, et al. Time-Restricted Eating Effects on Body Composition and Metabolic Measures in Humans who are Overweight: A Feasibility Study. Obesity (Silver Spring). 2020;28(5):860-9. <http://dx.doi.org/10.1002/oby.22756>. PMID: 32270927.

8. Cienfuegos S, Gabel K, Kalam F, Ezpeleta M, Wiseman E, Pavlou V, et al. Effects of 4- and 6-h Time-Restricted Feeding on Weight and Cardiometabolic Health: A Randomized Controlled Trial in Adults with Obesity. Cell Metab. 2020;32(3):366-78 e3. <http://dx.doi.org/10.1016/j.cmet.2020.06.018>. PMID: 32673591.

9. Wilkinson MJ, Manoogian EN, Zadourian A, Lo H, Fakhouri S, Shoghi A, et al. Ten-hour time-restricted eating reduces weight, blood pressure, and atherogenic lipids in patients with metabolic syndrome. Cell metabolism. 2020;31(1):92-104. e5. <http://dx.doi.org/10.1016/j.cmet.2019.11.004>. PMID: 31813824.

10. Lowe DA, Wu N, Rohdin-Bibby L, Moore AH, Kelly N, Liu YE, et al. Effects of Time-Restricted Eating on Weight Loss and Other Metabolic Parameters in Women and Men With Overweight and Obesity: The TREAT Randomized Clinical Trial. JAMA Intern Med. 2020;180(11):1491-9. <http://dx.doi.org/10.1001/jamainternmed.2020.4153>. PMID: 32986097.

11. Moon S, Kang J, Kim SH, Chung HS, Kim YJ, Yu JM, et al. Beneficial Effects of Time-Restricted Eating on Metabolic Diseases: A Systemic Review and Meta-Analysis. Nutrients. 2020;12(5):1267. <http://dx.doi.org/10.3390/nu12051267>. PMID: 32365676.

12. Andreato LV, Esteves JV, Coimbra DR, Moraes AJP, de Carvalho T. The influence of high-intensity interval training on anthropometric variables of adults with overweight or obesity: a systematic review and network meta-analysis. Obes Rev. 2019;20(1):142-55. <http://dx.doi.org/10.1111/obr.12766>. PMID: 30450794.

13. Gillen JB, Gibala MJ. Is high-intensity interval training a time-efficient exercise strategy to improve health and fitness? Applied physiology, nutrition, and metabolism. 2014;39(3):409-12.

14. Sharp T, Grandou C, Coutts AJ, Wallace L. The Effects of High-Intensity Multimodal Training in Apparently Healthy Populations: A Systematic Review. Sports medicine-open. 2022;8(1):1-16. <http://dx.doi.org/10.1186/s40798-022-00434-x>. PMID: 35348924.

15. Feito Y, Heinrich KM, Butcher SJ, Poston WSC. High-Intensity Functional Training (HIFT): Definition and Research Implications for Improved Fitness. Sports (Basel). 2018;6(3):76. <http://dx.doi.org/10.3390/sports6030076>. PMID: 30087252.

16. Nieuwoudt S, Fealy CE, Foucher JA, Scelsi AR, Malin SK, Pagadala M, et al. Functional high-intensity training improves pancreatic beta-cell function in adults with type 2 diabetes. Am J Physiol Endocrinol Metab. 2017;313(3):E314-E20. <http://dx.doi.org/10.1152/ajpendo.00407.2016>. PMID: 28512155.

17. Fealy CE, Nieuwoudt S, Foucher JA, Scelsi AR, Malin SK, Pagadala M, et al. Functional high-intensity exercise training ameliorates insulin resistance and cardiometabolic risk factors in type 2 diabetes. Exp Physiol. 2018;103(7):985-94. <http://dx.doi.org/10.1113/EP086844>. PMID: 29766601.

18. Fisher J, Sales A, Carlson L, Steele J. A comparison of the motivational factors between CrossFit participants and other resistance exercise modalities: a pilot study. J Sports Med Phys Fitness. 2017;57(9):1227-34.

19. Heinrich KM, Patel PM, O'Neal JL, Heinrich BS. High-intensity compared to moderate-intensity training for exercise initiation, enjoyment, adherence, and intentions: an intervention study. BMC Public Health. 2014;14(1):789. <http://dx.doi.org/10.1186/1471-2458-14-789>. PMID: 25086646.

20. Lu Y, Wiltshire HD, Baker JS, Wang Q. The Effects of Running Compared with Functional High-Intensity Interval Training on Body Composition and Aerobic Fitness in Female University Students. Int J Environ Res Public Health. 2021;18(21):11312. <http://dx.doi.org/10.3390/ijerph182111312>. PMID: 34769831.

21. Haganes KL, Silva CP, Eyjolfsdottir SK, Steen S, Grindberg M, Lydersen S, et al. Time-restricted eating and exercise training improve HbA1c and body composition in women with overweight/obesity: A randomized controlled trial. Cell Metab. 2022;34(10):1457-71 e4. <http://dx.doi.org/10.1016/j.cmet.2022.09.003>. PMID: 36198292.

22. Kotarsky CJ, Johnson NR, Mahoney SJ, Mitchell SL, Schimek RL, Stastny SN, et al. Time-restricted eating and concurrent exercise training reduces fat mass and increases lean mass in overweight and obese adults. Physiol Rep. 2021;9(10):e14868. <http://dx.doi.org/10.14814/phy2.14868>. PMID: 34042299.

23. Tabata I, Nishimura K, Kouzaki M, Hirai Y, Ogita F, Miyachi M, et al. Effects of moderate-intensity endurance and high-intensity intermittent training on anaerobic capacity and VO~ 2~ m~ a~ x. Medicine and science in sports and exercise. 1996;28:1327-30.

24. Borg G. Borg's perceived exertion and pain scales: Human kinetics; 1998.

25. Friedewald WT, Levy RI, Fredrickson DS. Estimation of the concentration of low-density lipoprotein cholesterol in plasma, without use of the preparative ultracentrifuge. Clin Chem. 1972;18(6):499-502. PMID: 4337382.

26. Wallace TM, Levy JC, Matthews DR. Use and abuse of HOMA modeling. Diabetes care. 2004;27(6):1487-95. <http://dx.doi.org/10.2337/diacare.27.6.1487>. PMID: 15161807.

27. Cohen J. Statistical power analysis. Current directions in psychological science. 1992;1(3):98-101.

28. Gabel K, Hoddy KK, Haggerty N, Song J, Kroeger CM, Trepanowski JF, et al. Effects of 8-hour time restricted feeding on body weight and metabolic disease risk factors in obese adults: A pilot study. Nutr Healthy Aging. 2018;4(4):345-53. <http://dx.doi.org/10.3233/NHA-170036>. PMID: 29951594.

29. Kang J, Ratamess NA, Faigenbaum AD, Bush JA, Beller N, Vargas A, et al. Effect of Time-Restricted Feeding on Anthropometric, Metabolic, and Fitness Parameters: A Systematic Review. J Am Nutr Assoc. 2022;41(8):810-25. <http://dx.doi.org/10.1080/07315724.2021.1958719>. PMID: 34491139.

30. Correia JM, Santos I, Pezarat-Correia P, Minderico C, Mendonca GV. Effects of Intermittent Fasting on Specific Exercise Performance Outcomes: A Systematic Review Including Meta-Analysis. Nutrients. 2020;12(5):1390. <http://dx.doi.org/10.3390/nu12051390>. PMID: 32408718.

31. Bhutani S, Klempel MC, Kroeger CM, Trepanowski JF, Varady KA. Alternate day fasting and endurance exercise combine to reduce body weight and favorably alter plasma lipids in obese humans. Obesity (Silver Spring). 2013;21(7):1370-9. <http://dx.doi.org/10.1002/oby.20353>. PMID: 23408502.

32. Maaloul R, Marzougui H, Dhia IB, Ghroubi S, Tagougui S, Kallel C, et al. Effectiveness of Ramadan diurnal intermittent fasting and concurrent training in the management of obesity: is the combination worth the weight? Nutrition, Metabolism and Cardiovascular Diseases. 2022.

33. Cooke MB, Deasy W, Ritenis EJ, Wilson RA, Stathis CG. Effects of Intermittent Energy Restriction Alone and in Combination with Sprint Interval Training on Body Composition and Cardiometabolic Biomarkers in Individuals with Overweight and Obesity. Int J Environ Res Public Health. 2022;19(13):7969. <http://dx.doi.org/10.3390/ijerph19137969>. PMID: 35805627.

34. Vieira AF, Costa RR, Macedo RCO, Coconcelli L, Kruel LFM. Effects of aerobic exercise performed in fasted v. fed state on fat and carbohydrate metabolism in adults: a systematic review and meta-analysis. British Journal of Nutrition. 2016;116(7):1153-64. <http://dx.doi.org/10.1017/S0007114516003160>. PMID: 27609363.

35. Anton SD, Moehl K, Donahoo WT, Marosi K, Lee SA, Mainous AG, 3rd, et al. Flipping the Metabolic Switch: Understanding and Applying the Health Benefits of Fasting. Obesity (Silver Spring). 2018;26(2):254-68. <http://dx.doi.org/10.1002/oby.22065>. PMID: 29086496.

36. Vasim I, Majeed CN, DeBoer MD. Intermittent Fasting and Metabolic Health. Nutrients. 2022;14(3):631. <http://dx.doi.org/10.3390/nu14030631>. PMID: 35276989.

37. Aird TP, Davies RW, Carson BP. Effects of fasted vs fed-state exercise on performance and post-exercise metabolism: A systematic review and meta-analysis. Scand J Med Sci Sports. 2018;28(5):1476-93. <http://dx.doi.org/10.1111/sms.13054>. PMID: 29315892.

38. Cassidy S, Thoma C, Houghton D, Trenell MI. High-intensity interval training: a review of its impact on glucose control and cardiometabolic health. Diabetologia. 2017;60(1):7-23. <http://dx.doi.org/10.1007/s00125-016-4106-1>. PMID: 27681241.
