# Supplementary material for: Correction: Unlocking the power of synergy: High-intensity functional training and early time-restricted eating for transformative changes in body composition and cardiometabolic health in inactive women with obesity
Source: PLoS One. 2024 May 31;19(5):e0305040. doi: 10.1371/journal.pone.0305040 (PMC11142490; doi:10.1371/journal.pone.0305040)
Supplement: S1 File — This file includes supplementary data. (PDF) [file pone.0305040.s001.pdf]

participant	groupe	height	weight-pre	weight-post	BMI pre	BMI post
**1**	TRF + EX	1,64	117	99	43,50	36,81
**2**	TRF + EX	1,71	92	81	31,46	27,70
**3**	TRF + EX	1,7	93,5	82	32,35	28,37
**4**	TRF + EX	1,7	97	90	33,56	31,14
**5**	TRF + EX	1,67	100	90	35,86	32,27
**6**	TRF + EX	1,64	88	79	32,72	29,37
**7**	TRF + EX	1,57	92	80	37,32	32,46
**8**	TRF + EX	1,65	85	77	31,22	28,28
**9**	TRF + EX	1,65	95	84	34,89	30,85
**10**	TRF + EX	1,68	93	77	32,95	27,28
**11**	TRF + EX	1,73	102	92	34,08	30,74
**12**	TRF + EX	1,63	104	97	39,14	36,51
**13**	TRF + EX	1,56	86	61	35,34	25,07
**14**	TRF + EX	1,65	110	97	40,40	35,63
**15**	TRF + EX	1,6	83	75	32,42	29,30
**16**	TRF + EX	1,73	98	87	32,74	29,07
**17**	TRF + EX	1,65	92	86	33,79	31,59
**18**	TRF + EX	1,6	85	76	33,20	29,69
**19**	TRF + EX	1,65	92	83	33,79	30,49
**20**	TRF + EX	1,64	119	100	44,24	37,18
**1**	TRF	1,59	101	95	39,95	37,58
**2**	TRF	1,67	86	80	30,84	28,69
**3**	TRF	1,65	117	96	42,98	35,26
**4**	TRF	1,67	87	80	31,20	28,69
**5**	TRF	1,67	88	85	31,55	30,48
**6**	TRF	1,63	87	74	32,74	27,85
**7**	TRF	1,57	92	88	37,32	35,70
**8**	TRF	1,65	85	72	31,22	26,45
**9**	TRF	1,63	87	75	32,74	28,23
**10**	TRF	1,57	96	80	38,95	32,46
**11**	TRF	1,7	108	97	37,37	33,56
**12**	TRF	1,55	81	80	33,71	33,30
**13**	TRF	1,55	77	65	32,05	27,06
**14**	TRF	1,68	92	85	32,60	30,12
**15**	TRF	1,59	87	82	34,41	32,44
**16**	TRF	1,59	83	72	32,83	28,48
**17**	TRF	1,7	131	120	45,33	41,52
**18**	TRF	1,57	77	74	31,24	30,02
**19**	TRF	1,69	112	98	39,21	34,31
**20**	TRF	1,58	82	79	32,85	31,65
**1**	EX	1,64	83	77	30,86	28,63

**2**	EX	1,62	85	74	32,39	28,20
**3**	EX	1,6	106	103	41,41	40,23
**4**	EX	1,7	115	112	39,79	38,75
**5**	EX	1,65	88	86	32,32	31,59
**6**	EX	1,55	90	87	37,46	36,21
**7**	EX	1,57	91	86	36,92	34,89
**8**	EX	1,51	96	90	42,10	39,47
**9**	EX	1,55	81	79	33,71	32,88
**10**	EX	1,56	80	68	32,87	27,94
**11**	EX	1,66	86	74	31,21	26,85
**12**	EX	1,7	89	77	30,80	26,64
**13**	EX	1,52	78	73	33,76	31,60
**14**	EX	1,65	107	104	39,30	38,20
**15**	EX	1,56	81	75	33,28	30,82
**16**	EX	1,51	77	75	33,77	32,89
**17**	EX	1,62	93	87	35,44	33,15
**18**	EX	1,58	82	80	32,85	32,05
**19**	EX	1,6	97	89	37,89	34,77
**20**	EX	1,6	83	80	32,42	31,25
**21**	EX	1,59	101	98	39,95	38,76
**22**	EX	1,55	75	70	31,22	29,14
**23**	EX	1,6	85	77	33,20	30,08
**24**	EX	1,67	87	85	31,20	30,48

WC pre	WC post	HC pre	HC post	WHR pre	WHR post
110	100	131	118	0,84	0,85
112	97	122	109	0,92	0,89
104	95	122	110	0,85	0,86
95	87	117	106	0,81	0,82
108	98	120	115	0,90	0,85
95	88	118	107	0,81	0,82
106	95	122	115	0,87	0,83
102	97	118	109	0,86	0,89
105	95	120	110	0,88	0,86
92	87	119	109	0,77	0,80
105	89	121	111	0,87	0,80
105	97	119	104	0,88	0,93
114	96	112	102	1,02	0,94
119	107	130	120	0,92	0,89
90	87	113	106	0,80	0,82
98	87	123	113	0,80	0,77
95	88	118	106	0,81	0,83
104	90	125	105	0,83	0,86
107	88	119	112	0,90	0,79
111	98	134	121	0,83	0,81
101	97	123	119	0,82	0,82
98	93	119	105	0,82	0,89
100	95	133	122	0,75	0,78
90	87	120	115	0,75	0,76
90	87	112	106	0,80	0,82
88	70	115	105	0,77	0,67
110	100	121	115	0,91	0,87
101	97	112	112	0,90	0,87
93	87	105	117	0,89	0,74
104	98	112	101	0,93	0,97
132	117	114	112	1,16	1,04
91	84	123	110	0,74	0,76
94	90	103	100	0,91	0,90
102	95	115	122	0,89	0,78
101	97	113	109	0,89	0,89
95	90	113	105	0,84	0,86
103	92	143	133	0,72	0,69
90	83	109	106	0,83	0,78
105	100	134	119	0,78	0,84
90	85	108	100	0,83	0,85
112	107	118	111	0,95	0,96

96	87	123	97	0,78	0,90
115	104	134	130	0,86	0,80
127	116	120	116	1,06	1,00
93	88	112	110	0,83	0,80
95	87	119	115	0,80	0,76
114	110	124	118	0,92	0,93
115	106	117	113	0,98	0,94
102	93	120	115	0,85	0,81
97	91	115	100	0,84	0,91
96	87	124	115	0,77	0,76
100	94	117	105	0,85	0,90
98	90	120	115	0,82	0,78
108	100	122	126	0,89	0,79
110	106	106	99	1,04	1,07
107	100	117	109	0,91	0,92
107	95	118	111	0,91	0,86
95	90	118	114	0,81	0,79
111	102	124	121	0,90	0,84
106	97	118	113	0,90	0,86
97	93	123	119	0,79	0,78
95	88	117	113	0,81	0,78
102	95	114	111	0,89	0,86
99	88	122	120	0,81	0,73

Fat mass pre	Fat mass post	FFM pre
55,9	48,2	21,4
37	28	23,9
36,8	34,4	20,4
41,9	28	25,1
44,7	35,7	24
36,4	31,2	20,4
38,2	27,3	25,4
26,8	24,3	30,4
41,4	31	20,8
26,8	25	23,1
49,9	32,1	21,9
47,4	35	25,2
25,8	19,5	19,6
43,6	35,2	26
24,9	23	22
39,8	35,2	27,6
37,3	28,9	23,7
32,6	24,5	20,1
41,8	33	19,2
57,2	41,3	25,2
42,1	40,2	23
39,2	38	33
45,7	36,2	26,2
30,4	28,2	28,6
37,2	32,1	24,3
16,3	15,6	27,1
40,6	38,2	30,8
32,9	30,6	29
28,4	25,3	21,2
33,9	30,2	24,4
44,6	38,3	23
36,3	35,6	29
26,1	24,6	25,3
40,2	35,8	23,3
37	33,9	23,3
26,5	26,3	23
43,4	30,4	27,8
34,3	31,2	19,6
48	35,7	19,7
29,7	25,4	20,3
36,3	34	21,3

24	20,8	19,7
52	48,2	19,3
55,2	50	26,2
34,7	33	21,8
38	34,5	22,5
37,9	35,8	22,5
46	43	21,1
37,8	37,5	19,5
23,5	20,3	22,3
28,6	26,6	21,3
23	20	30
32,6	29,5	20,8
47,3	46,4	28,4
28,1	25,3	18,6
30,7	28	20,3
37	31	25
28,9	25,3	25,7
43,9	38,4	19,7
35,8	33,4	22
43	40,3	25,7
27,7	23,1	23,2
36,4	35,9	16,1
30,1	29,9	18,3

FFM post	glucose pre	glucose post	cholesterol pre
27	4,6	3,9	4,54
26,2	5,2	4,4	4,39
25,7	3,8	4	4,35
31,9	5,9	3,4	7,53
26,7	5,5	4,9	6,3
24,4	5,4	4,9	4
31,2	4,4	2,8	5,15
34,2	5,6	4,2	4,61
24,3	3,5	3	6,63
26,3	5,9	3,9	4,01
24,8	4,9	3,9	4,78
29,8	5,8	3,8	5,84
21,6	4,2	2,2	4,82
27,8	4,2	4	5,09
26,4	4,2	2	4,31
30,2	5,7	4,4	4,71
26,3	5,7	4,4	6,64
24,5	4,8	4,3	4,41
22,2	4,4	2,3	6,89
25,8	5,4	3,7	5,18
21,9	3,6	2,8	4,67
20,9	4,6	4,2	3,81
24	5	3,06	4,56
32,1	5,3	4,9	4,43
27,1	4,9	5,3	4,69
29	4,9	5,3	4,91
23,2	5,4	4,4	6,15
22	5,6	3,5	3,53
24,7	4	2,5	3,03
23	4,4	3,4	6,87
27,1	4,4	3,4	5,62
21,2	5,2	4,7	3,55
20,8	3,7	5,1	5,89
24,8	4,3	2,6	4,38
23,8	5	3	4,99
22,5	5,1	3,8	4
29,4	4,5	3,8	4,61
20,3	4,8	3,4	3,47
18,9	5,4	3,1	5,06
25,4	4,3	3,1	5,03
22	6	4,6	4,56

21,7	5,5	4,7	5,53
24,4	5,2	4,4	5,74
29,2	6,2	4,3	5,13
25,4	5,1	4,7	3,51
24,8	4,7	5,1	4,47
23,5	5,4	4,8	4,66
26,4	6,6	5,3	4,67
24,1	5,7	4,7	4,05
26,8	3,8	3,6	4,85
23,6	3,8	3,6	3,91
33,5	5	4,1	4,67
25,6	4,8	3,8	6,47
30,4	4,4	4,9	4,46
20,1	5,3	4,7	7,37
22	5,5	4,6	6,24
26,4	5,5	4,6	5,55
27,9	5,8	4,8	6,3
21,7	5,4	4,8	5,6
25,4	5,7	5,8	3,99
29,4	5,9	4,4	3,73
24,9	5,8	4,7	6,13
21,6	5,2	4,9	4,13
23,5	5,6	4	5,79

cholesterol post	TG pre	TG post	HDL pre	HDL post	LDL pre	LDL post
3,85	0,81	0,74	1,18	2,65	2,99	0,86
3,28	0,91	0,72	1,29	2,48	2,69	0,47
3,5	0,51	0,12	1,07	2	2,31	1,8
4,2	2,14	0,53	1,39	2,3	3,17	0,78
4,25	0,79	0,78	1,54	2,15	3,54	1,75
3,37	1,8	0,56	1,29	2,59	1,89	0,53
3,6	0,94	0,78	1,06	2,3	3,74	0,96
3,24	1,86	0,94	1,26	1,76	2,5	2
4,38	1,86	0,62	1,32	2,62	2,95	1,75
3,04	0,79	0,72	1,27	2,4	2,46	2,03
3,81	0,62	0,56	1,06	2,01	3,33	1,89
4,13	0,86	0,65	1,26	2,53	3,91	2,9
4,42	2,24	1,98	1,32	1,8	2,9	2,16
3,89	0,66	0,56	1,27	2,5	4,72	2,84
3,2	3,03	1,15	1,06	2,6	2,64	1,8
3,73	1,05	0,87	1,03	2,43	2,9	1,45
4,12	2,12	0,77	1,61	2,56	2,72	2,23
3,59	1,5	1,23	0,99	1,19	2,43	0,78
3,38	1,6	0,84	1,19	2,76	2,52	1,49
4,01	0,85	0,73	1,46	2	5,25	2,26
3,49	1,23	0,98	1,41	1,78	2,7	1,26
3,37	0,99	0,84	1,1	1,88	2,26	1,11
3,2	1,56	1,21	0,93	1,39	2,92	1,38
3,78	2,96	2,77	1,25	1,8	2,74	1,63
4,57	0,7	0,74	1,16	2,76	2,21	1,47
4,47	0,66	0,53	1,74	1,39	2,87	2,84
5,01	1,35	1,05	1,55	1,8	3,99	0,98
4,57	1,35	1,05	1,11	2,83	2,23	1,3
2,99	0,42	0,84	1,35	1,52	3,46	3,31
5,94	0,49	0,46	2,08	0,65	4,22	2,63
4,49	1,26	1,05	0,62	1,7	3,67	2,08
3,31	2,92	1,96	1,45	2,09	3,9	5,32
5	0,45	0,75	1,45	2,42	2,05	1,5
4,46	0,85	1,8	1,05	2,28	2,93	1,99
4,42	0,88	0,84	1,18	2,21	3,39	1,54
3,96	2,93	3,01	0,81	0,86	2,59	1,37
3,03	1,32	0,69	1,01	2,21	3	0,48
2,96	1,33	0,75	1,14	1,41	2,04	1,86
4,78	0,82	0,58	1,01	2,83	3,46	2
5,08	1,02	1,25	1,5	1,2	4,75	4,31
4	2,1	1,8	1,26	1,38	5	3

3,63	2,01	1,5	1,34	2,11	2,49	0,84
5,74	1,32	0,88	1,71	3,51	3,43	1,83
3,77	1,87	2,08	1,04	1,54	3,24	1,28
3,66	0,85	0,89	1,04	1,44	2,08	1,82
3,94	1,35	0,98	1,03	2,48	2,83	1,01
3,65	0,93	0,77	1,33	1,54	2,91	1,67
4,57	0,93	0,77	1,85	2,5	2,23	1,74
2,92	1,17	0,79	1,35	1,97	2,29	0,52
3,45	0,91	0,72	1,65	2,13	2,83	1,6
3,08	0,82	1,12	1,02	2,13	2,44	0,8
4,8	0,99	0,68	1,44	2,78	2,72	2,33
6,36	1,12	0,75	1,58	2,24	4,39	3,84
4,55	1,11	0,85	1,05	3	2,75	1,01
5,81	1,46	1,68	1,52	1,81	5,22	3,05
3,22	1,38	1,15	1,68	2,36	3,76	2,24
4,5	1,75	0,9	1,21	3,22	3,74	2,06
4,65	1,31	1,39	1,58	1,19	3,98	2,5
5,29	1,63	1,4	2,17	2,32	2,86	1,59
5,78	1,25	1,05	1,12	2,25	2,41	0,9
3,22	1,02	1,9	1,34	1,52	2,14	0,64
5,32	0,55	0,57	1,13	1,06	4,63	3,4
4,63	1,2	0,9	1,21	2,13	2,52	2,77
4,64	2,21	0,74	1,44	1,65	3,96	3,28

insulin pre µUI/mL	insulin post µUI/mL	HOMA-IR pre	HOMA-IR post	PAS pre
8,8	4	1,80	0,69	125
10,1	6,5	2,33	1,27	120
12,5	2,1	2,11	0,37	120
12,5	3	3,28	0,45	130
7,1	4,8	1,74	1,05	120
21	3,6	5,04	0,78	110
14	11	2,74	1,37	125
8,2	6,5	2,04	1,21	125
16,2	15	2,52	2,00	120
17,3	7	4,54	1,21	115
8,4	7,4	1,83	1,28	125
23,7	8,5	6,11	1,44	135
13	2,9	2,43	0,28	110
22,7	5,2	4,24	0,92	125
7,1	8,7	1,33	0,77	120
12,8	9,5	3,24	1,86	125
12,1	6,9	3,07	1,35	120
16	5	3,41	0,96	120
6,8	6,5	1,33	0,66	110
19,9	10,9	4,78	1,79	125
6,8	6,9	1,09	0,86	120
14,7	14,7	3,01	2,74	140
20,8	6,7	4,62	0,91	120
17,2	18,4	4,05	4,01	120
17,2	5,8	3,75	1,37	115
16,2	14,4	3,53	3,39	120
7,5	5,6	1,80	1,10	120
9,6	3,3	2,39	0,51	120
11	15,2	1,96	1,69	125
18,6	9,2	3,64	1,39	115
18,9	8,7	3,70	1,31	110
15,3	15,8	3,54	3,30	125
11,3	7,8	1,86	1,77	150
11,5	9	2,20	1,04	110
8,2	5	1,82	0,67	125
9,3	8,4	2,11	1,42	135
17,9	17,1	3,58	2,89	120
11,3	14,8	2,41	2,24	125
8,3	9,4	1,99	1,30	120
15,7	14	3,00	1,93	100
8,6	4,9	2,29	1,00	120

14,7	11,03	3,59	2,30	125
10,8	4,7	2,50	0,92	110
23	23	6,34	4,40	110
8,3	9,8	1,88	2,05	110
11,5	7,7	2,40	1,75	115
7,2	3,2	1,73	0,68	110
8,5	6,2	2,49	1,46	125
15,9	17,3	4,03	3,61	120
21	17,9	3,55	2,86	125
8,5	4,7	1,44	0,75	140
17,2	15,3	3,82	2,79	130
15,9	18,9	3,39	3,19	125
15	14,9	2,93	3,24	140
8,5	2,7	2,00	0,56	120
14,3	12,5	3,50	2,56	145
9,4	4,4	2,30	0,90	130
19,5	11,8	5,03	2,52	140
15,3	15,7	3,67	3,35	125
14,9	17,6	3,77	4,54	140
7,8	5,2	2,05	1,02	135
9,3	5,6	2,40	1,17	120
16,3	5	3,77	1,09	135
17,6	14,1	4,38	2,51	120

PAS post	PAD pre	PAD post				
110	70	70				
120	70	70				
120	70	70				
125	70	70				
100	70	60				
110	70	70				
120	85	80				
120	75	70				
110	80	70				
110	85	80				
125	70	70				
130	75	70				
100	70	70				
120	75	75				
115	70	70				
115	75	70				
110	80	70				
120	85	80				
115	70	75				
115	80	70				
120	75	70				
130	80	75				
115	70	75				
110	70	70				
120	75	75				
120	80	70				
125	75	80				
110	80	70				
125	60	60				
125	70	80				
110	70	70				
125	75	80				
140	80	80				
110	80	70				
120	70	70				
130	85	80				
115	70	60				
130	60	70				
120	80	75				
115	65	75				
110	80	70				

120	85	80				
120	70	80				
110	75	70				
110	70	70				
110	75	75				
100	70	60				
115	70	70				
120	70	75				
120	75	70				
135	70	70				
100	70	70				
110	70	70				
120	80	75				
110	80	70				
130	80	75				
125	75	70				
130	60	70				
115	75	75				
120	80	70				
130	75	70				
120	80	75				
125	85	75				
120	75	70				

